# Dysfunctional information processing in individuals with acute exposure to sexual abuse: An ERP study

**DOI:** 10.1097/MD.0000000000010880

**Published:** 2018-06-01

**Authors:** Changwoo Han, Minkyung Park, Jun-Young Lee, Hee Yeon Jung, Su Mi Park, Jung-Seok Choi

**Affiliations:** a Department of Psychiatry, Eulji University Gangnam Eulji Hospital, Seoul; b Department of Addiction Rehabilitation Social Welfare, Eulji University, Kyunggi; c Department of Psychiatry, SMG-SNU Boramae Medical Center; d Department of Psychiatry and Behavioral Science; e Department of Clinical Medical Sciences, Seoul National University College of Medicine, Seoul, Republic of Korea.

**Keywords:** acute stress disorder (ASD), auditory oddball task, event-related potential (ERP), P300, posttraumatic stress disorder (PTSD)

## Abstract

Acute stress disorder (ASD) and posttraumatic stress disorder (PTSD) may occur after traumatic event and also cause significant life time impairment. P300 event-related potential (ERP) is a potential biological marker for PTSD and can reflect cognitive impairment in information processing and attention. Despite the usefulness of ERP, there are few attempts to reveal relationships between ASD and P300. In the present study, we aimed to determine if the P300 of the patients who were the victims of sexual abuse reflected the quantitative trait of ASD or if P300 is applicable as a state marker for predicting the risk of PTSD.

Fifteen female victims of sexual abuse diagnosed with ASD and 18 healthy controls (HCs) without trauma exposure participated in this study. We investigated the P300 ERPs in patients with ASD to compare them with those of HCs. ERPs were acquired from female adults during an auditory oddball task. Between-group differences in amplitudes or latencies of P300 were investigated using repeated-measures analysis of variance.

The ASD groups showed reduced P300 amplitudes at the midline centroparietal site as well as reduced accuracy rates during an auditory oddball task compared with the HCs.

These results indicate that ASD have abnormalities in the P300 compared to those in HCs. Moreover, the reduction in P300 could be considered a candidate neurophysiological marker for ASD.

## 1 Introduction

Acute stress disorder (ASD) and posttraumatic stress disorder (PTSD) may occur after traumatic events and may cause significant life-time impairment. Symptoms of ASD present from 2 days to 4 weeks after trauma and include dissociative, reexperiencing, avoidance, and hyperarousal symptoms.^[1]^ ASD differs from PTSD with respect to time course and has a greater emphasis on dissociative symptoms, such as numbing, reduced awareness, derealization, depersonalization, and dissociative amnesia.^[1]^ ASD symptoms and diagnoses may be the closest data to predict PTSD. One study reported that ASD patients would be very likely to suffer from PTSD.^[2]^ Another study found that the dissociative, reexperiencing, avoidant, or arousal criteria for ASD diagnosis could have important predictive power for the development in PTSD.^[3]^ However, the predictability of the change from ASD to PTSD in trauma samples is still controversial.

The life-time prevalence of PTSD has been reported to reach 6.8%. However, in those exposed to trauma, the lifetime prevalence was significantly higher, at 15%.^[4,5]^ Moreover, the prevalence of PTSD among females has been reported to be twice as high as in males.^[6]^ Compared with men, women are more likely to be exposed to assault and rape. In such cases, female victims tended to experience PTSD symptoms 5 times more commonly.^[7]^ Indeed, there are 3 postrape symptoms, according to DSM-5, defining PTSD: reexperience of the traumatic event, avoidance, and hyperarousal symptom.^[1]^ A previous study reported that 76% of rape victims met the symptom criteria for PTSD within 1 year after the assault.^[8]^ Another study showed that 94% of rape victims met the diagnostic criteria for PTSD at the point of the assault, and 47% continued to have symptoms of PTSD 3 months later.^[7]^ Thus, if we can discover biological markers associated with ASD, this may be of great help in discovering PTSD earlier.

There have been many efforts to establish biological markers for ASD and PTSD. Gamma amino-butyric acid (GABA) plasma levels in the brain were found to be low after trauma exposure.^[9]^ This could suggest that individuals with low GABA plasma levels may be at a higher risk of developing ASD and PTSD. Heart rate (HR) measured after a physical trauma can predict PTSD.^[10]^ Having a diagnosis of ASD or a heightened resting HR has been demonstrated to be associated with PTSD symptoms 2 years later.^[11]^ The cortisol level of rape victims in the emergency room is a predictor of PTSD and depression.^[12]^ Moreover, a neuroimaging-derived endophenotype, developed with functional magnetic resonance imaging, has been found to react to traumatic cues.^[13]^

The P300 component of an event-related potential (ERP) is another possible biological marker for PTSD.^[14]^ However, little research has explored symptoms of ASD, especially in victims of rape or sexual assault, using ERP techniques. ERP can reflect information processing by stimulus or task. ASD and PTSD subjects showed cognitive impairment in information processing and attention deficit.^[15,16]^ Thus, P300 may be a useful tool to assess information processing in ASD and PTSD subjects.

Although various studies have been attempted to reveal relationships between PTSD and P300, there are few studies on the correlation between ASD (as a predictor of PTSD) and P300. In the present study, we aimed to determine whether the P300 of patients who were victims of sexual abuse reflected the quantitative traits of ASD and whether P300 may be applicable as a state marker for predicting the risk of PTSD.

## 2 Materials and methods

### 2.1 Participants

This prospective study included a total of 33 young females: 15 subjects who had been sexually abused (age: 25.41 ± 8.93 years) who presented to the Emergency Department at SMG-SNU Boramae Medical Center, Seoul, Korea, after the abuse, and 18 healthy controls (age: 23.32 ± 2.86 years) without trauma exposure. Female victims were referred to psychiatrists within 2 weeks after the incident and diagnosed with ASD according to DSM-5. Additionally, the Structured Clinical Interview for DSM-IV disorders (SCID) was used to identify past and current psychiatric illnesses.^[17]^ All subjects underwent a baseline evaluation by electroencephalography (EEG), as well as the Beck Depression Inventory (BDI)^[18]^ and the Beck Anxiety Inventory (BAI)^[19]^ within 4 weeks postincident. In order to investigate the development of PTSD, a clinical interview by experienced psychiatrists was administered for the diagnosis of PTSD, according to DSM-5, at 12 weeks postincident. They were also assessed at 12 weeks postincident using the Clinician-Administered Post-traumatic Stress Disorder Scale (CAPS).^[20]^

The healthy controls (HC) were recruited from the local community and had no history of any psychiatric disorder. Participants, regardless of group, with a history of head injury, seizure disorder, psychotic disorder, mental retardation, or substance use disorder, except nicotine, were excluded. All participants were medication-naïve at the time of assessment.

The institutional review board of the SMG-SNU Boramae Medical Center approved the study protocol. All subjects provided written informed consent.

### 2.2 Task and procedure

We used an auditory oddball task, which involved presenting standard stimuli (85%, 2000 Hz) and target stimuli (15%, 1000 Hz) in a pseudorandomized order. In total, 300 stimuli were delivered binaurally using a sound generator (STIM 2; Compumedics, El Paso, TX). Insert earphones were used to present the auditory stimuli. The loudness of the stimuli was 85 dB sound pressure level (SPL). All stimuli were presented for 100 milliseconds (including 10-millisecond rise and fall times) with fixed inter-trial intervals of 1250 milliseconds. The task required subjects to press a button with their right hand as quickly and accurately as possible only when target stimuli were presented. All participants were given the opportunity to practice before the actual task started. Participants completed 3 blocks of 100 trials while seated in a comfortable chair. A detailed description of the experimental procedure was described in our previous report.^[21]^

### 2.3 ERP recording

EEG and electrooculogram (EOG) data were recorded continuously using a 64-channel Quick-cap system (Compumedics) according to the extended international 10 to 20 system in a dimly lit, isolated sound-shielded room. The location of the ground channel was between FPz and Fz and the linked electrodes at the left and right mastoids served as reference electrodes. To monitor eye movements, vertical EOG was measured above and beneath the left eye and horizontal EOG was measured at the outer canthus of each eye. The electrical activities were recorded continuously at a sampling rate of 250 or 500 Hz. All data were processed with a 0.3 to 100 Hz band-pass filter. Impedance was below 10 kΩ.

### 2.4 ERP analysis

Electrophysiological signals were further processed off-line using the Curry 7 software (Compumedics). Recordings were first downsampled to 250 Hz. EEG data were then rereferenced to the common average reference and filtered between 0.3 and 30 Hz. Gross artifacts on EEG data such as those involving movement were visually inspected by an expert and rejected. Artifacts related to eye blinks and eye movements were corrected using the algorithm of artifact reduction developed by Semlitsch et al.^[22]^ Data were then segmented into epochs of 1000 milliseconds including the 100-millisecond before stimulus onset and 900 milliseconds after stimulus onset. Epochs containing EEG data that exceeded ± 85 μV were considered artifacts and discarded. Only artifact-free epochs and correctly responded trials to deviant tones at 3 midline sites (Cz, CPz, and Pz) were averaged and analyzed. The ERP waveforms for each participant had a minimum of 35 artifact-free trials. The P300 component was defined as the largest positive-going peak within the time window between 248 and 500 milliseconds poststimulus onset. Topographic maps were created using Matlab (ver. 7.10.0; MathWorks, Natick, MA) and the EEGLAB toolbox.^[23]^

### 2.5 Statistical analysis

Demographic, clinical, and behavioral data were analyzed with one-way analyses of variance (ANOVAs) to compare group differences. In terms of stimulus-locked ERP values, the amplitudes and latencies of the P300 component were analyzed separately with repeated-measures ANOVAs (rmANOVAs) with electrode sites (Cz, CPz, and Pz) as within-subject factors and group as the between-subjects factor. In cases of sphericity violations, lower-bound corrections were applied, and corrected *P* values are reported. P300 values associated with significant intergroup differences were subjected to analyses with clinical variables using 2-tailed Pearson correlation coefficients. Results with *P* values <.05 were regarded as significant. All statistical analyses were performed using the SPSS software (ver. 18.0; SPSS, Inc., Chicago, IL).

## 3 Results

### 3.1 Subject characteristics

Demographic and clinical characteristics of the participants are presented in Table 1. There was no significant difference in age or IQ between the ASD and HC groups, but the ASD patients had a lower education level (*P* = .017) than the HCs. The ASD group had higher BDI (*P* < .001) and BAI (*P* = .004) scores than the HC group. At 12 weeks after the sexual abuse incident, 8 of 15 (53.3%) ASD patients developed PTSD. Subjects with PTSD showed higher BDI scores at baseline than those without PTSD. There was no significant difference in BAI scores at baseline or CAPS scores at 12 weeks postincident between the PTSD and non-PTSD group, although the PTSD group had higher BAI and CAPS scores.

**Table 1:**
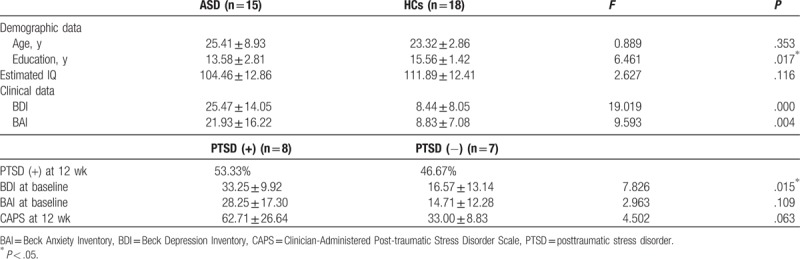
Demographic and clinical characteristics of patients with acute stress disorder (ASD) and healthy controls (HC).

### 3.2 Behavioral results

The ASD patients showed lower accuracy rates (*F*_(1, 31)_ = 6.923, *P* = .013) than the HC group. Although ASD patients showed somewhat delayed reaction time compared with HCs, no significant group difference was observed. Behavioral performance data are presented in Table 2.

**Table 2:**
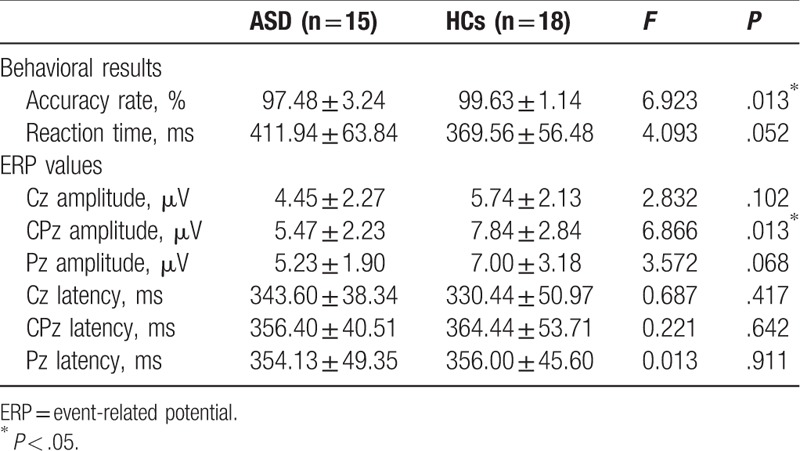
Behavioral results (accuracy rates and reaction times) and ERP values (amplitudes and latencies of P300) in patients with acute stress disorder (ASD) and healthy controls (HC).

### 3.3 P300 in subjects who had recently experienced an acute traumatic event

**Figure 1:**
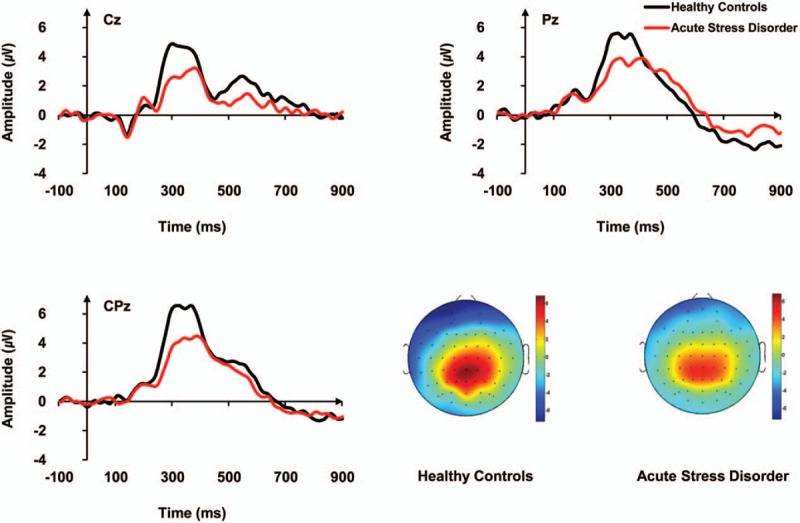
Grand-average ERP waveforms over 3 electrode regions (Cz, CPz, and Pz) in response to deviant tones in the auditory oddball task for patients with acute stress disorder (ASD) and healthy controls (HCs).

The grand-average ERP waveforms for deviant stimuli at the 3 electrode sites are shown in Fig. 1. Significant main effects of electrode site (*F*_(2, 62)_ = 6.962, *P* = .013) and group (*F*_(1, 31)_ = 6.308, *P* = .017) for P300 amplitudes were found. The P300 amplitude measured at CPz was higher than that at Cz. No significant interaction was observed between electrode site and group for P300 amplitudes. The ASD group showed significantly lower P300 amplitudes than HCs at CPz (*F*_(1,_._31)_ = 6.866, *P* = .013) but not at Cz or Pz. When education, BDI, and BAI were included as covariates to control the effects of education, BDI, and BAI on P300 amplitude at CPz, significant differences in P300 amplitudes between the groups remained (*F*_(1, 25)_ = 6.008, *P* = .022). None of the main effects, interactions, or group effect was statistically significant for P300 latencies.

### 3.4 P300 in subjects with and without PTSD

**Figure 2:**
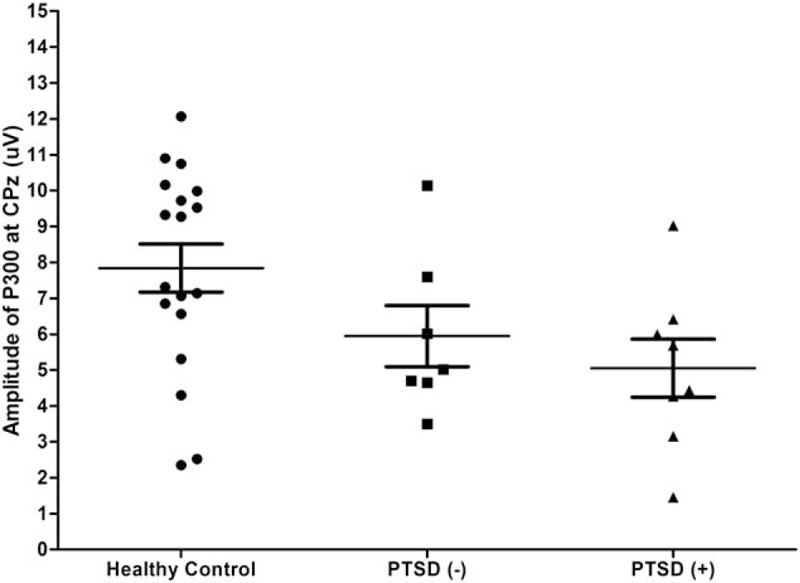
Scatter plot of P300 amplitudes at CPz during the auditory oddball task in healthy controls, and patients with and without posttraumatic stress disorder (PTSD).

Although PTSD patients showed somewhat lower P300 amplitudes at CPz than the non-PTSD group, no significant group difference in P300 measures at baseline was observed. P300 amplitudes at CPz are shown in Fig. 2.

### 3.5 Correlation of P300 with clinical symptoms

No significant correlation was observed between P300 amplitudes at the CPz site and BDI, BAI, or CAPS scores in individuals with ASD.

## 4 Discussion

This study found that the amplitude of P300, as a biological marker, was reduced significantly in patients diagnosed with ASD that developed within 1 month of sexual abuse incidents. This alteration in P300 was observed in patients diagnosed with ASD, a precursor stage of PTSD, as in PTSD patients. It is known that the amplitude of P300 is altered in PTSD patients.^[24]^ Some previous studies reported that patients with PTSD showed reduced amplitudes of P300 to trauma-neutral stimuli,^[25]^ whereas most findings have shown that patients with PTSD have enhanced P300 amplitudes in response to trauma-relevant stimuli.^[26]^

Unlike studies involving patients with PTSD, there have been few findings concerning cortical activities of patients with ASD, as assessed by measurements of EEG or ERP. According to Felmingham et al,^[24]^ the amplitudes of P300 components at a midline parietal site elicited by target stimuli in an auditory oddball task were higher in ASD patients, who were survivors of physical assault or motor vehicle accidents, than in PTSD patients and HCs.

However, to date, there has been no reported study investigating the characteristics of female patients with ASD after a sexual assault in the ERP domains. Given that P300 is a late cognitive component of ERPs and reflects neural activity related to working memory processes and attention,^[27]^ the finding that reduced amplitudes of P300 to neutral target stimuli in patients with ASD indicates information processing deficits in ASD patients, which is similar to previous studies related to the PTSD patients.^[28]^ Moreover, this finding supports previous studies in that patients with ASD showed impairment in memory, compared with HCs, and the severity of ASD symptoms were associated with delayed verbal memory.^[29–32]^ Given previous studies in which PTSD patients showed reduced P300 amplitudes to neutral target stimuli, the fact that the amplitude of P300 was reduced in patients with ASD might be due to auditory nontrauma related stimuli features and to the characteristics of the participants who were sexual assault female victims studied here.

Additionally, we investigated the occurrence of PTSD in patients with ASD to assess the relationship between ASD patients who developed PTSD versus those who did not. Follow-up assessments to confirm the diagnosis of PTSD were completed at 12 weeks from the time of the sexual assaults. We found no significant difference in P300 between PTSD and non-PTSD groups. However, the PTSD group showed a reduction in the average value that was more than that in the non-PTSD group. Thus, we might have obtained significant results in a study with more patients. In particular, the subjects of this study were victims of sexual abuse, and existing studies have shown that patients who have experienced sexual assaults versus other trauma were more likely to develop PTSD after the initial ASD.^[33]^ Thus, these findings suggest that P300 may be a useful biological marker for ASD, as it is for PTSD.

Most patients initially diagnosed with ASD developed PTSD, but often they develop other mental diseases, such as depressive symptoms and panic disorder.^[34]^ Moreover, the power of ASD to predict PTSD is high, but it has been proposed that its sensitivity is low-to-moderate.^[2]^ ASD must have 3 symptoms or more, among the 5 dissociative symptoms: slowdown of emotional reaction, reduction in surrounding environment recognition, derealization, depersonalization, and dissociative amnesia. However, there are differences in diagnostic criteria: it is possible to diagnose PTSD without dissociation. Such a difference in diagnostic criteria might be a reason for the 2 diseases not being considered on the same continuum. Nevertheless, many studies have revealed that ASD can predict PTSD in trauma patients, since its first introduction in DSM-IV. There are some studies that have overestimated the predictive power of ASD for PTSD. As a result of studying the predictive power of PTSD in the victims of violent crimes, overall symptom clusters could predict subsequent PTSD.^[35]^ Even a study on typhoon survivors showed that the patients diagnosed with ASD were more likely to be diagnosed with PTSD later.^[36]^ There is also a study that demonstrated that in cases of violent trauma, female ASD patients were more likely to develop PTSD than male patients.^[37]^ The study reported that in cases of ASD resulting from motorcycle accidents, significantly more female patients developed PTSD later than did males.

Looking at studies related to P300 and PTSD, patients with PTSD showed a significantly lower amplitude of P300 than the patients without PTSD.^[38]^ The lower P300 amplitude at Pz was significantly associated with higher avoidance/numbing scores in the PTSD group.^[38]^ Some studies subdivided P300 and analyzed the association with PTSD.^[39]^ P300a represents a “response to involuntary attention,” P300b an “association with voluntary attention,” and P300wm a “component associated with working memory.” According to a meta-analysis, there was a reduction in the amplitude of P300 in PTSD patients, and P300a increased in PTSD patients versus non-PTSD ones when a stimulus related to trauma was given. P300b also increased under the stimulus related to trauma. However, under a neutral stimulus, the amplitude of P300b was reduced in patient groups versus normal groups. The present study used the method to measure patients’ attention using a neutral stimulus and measured the overall amplitude of P300. That the amplitude among patient groups was reduced in this study is considered to be similar to previous studies.^[28]^

The prognosis of PTSD may lead to chronic effects. According to previous studies, PTSD can occur sustained medical illnesses such as diabetics and cardiovascular disease.^[40,41]^ And PTSD can lead to various severe disabilities, including academic failure, instability in marriage, and unemployment, as well as mental illnesses that result in overall impairment beyond the symptoms of anxiety disorder.^[42]^ PTSD may result in increased impulsivity, leading to suicide attempts, and may be associated with depression, anxiety disorders, and substance abuse. Thus, appropriate diagnosis and treatment of PTSD is important for preventing the prognosis in patients with chronic PTSD from deteriorating. As this study showed an association of P300 in ASD patients due to exposure to trauma, it will be helpful for assessing the possibility of ASD developing into PTSD later. Despite this clinical importance, the present study has some limitations. First, this study used a small number of subjects. Patients who participated in this study were restricted to female sexual abuse victims and were compared with a control group. Small sample size could have limitations to explain the relationships between ASD and P300. Although there are obvious limitations, this is a pilot study to reveal the predictability of P300 in ASD. Our results suggest P300 as a useful biological marker to predictor of ASD. If we recruit a larger number of subjects in further studies, the results will, hopefully, be clearer. Second, this study measured the overall amplitude of P300 using a neutral stimulus. If we had measured ERP using other stimuli, or made a detailed analysis through the subdivision of ERP, this might have been helpful in demonstrating a neurophysiological association between ASD and ERP. Despite these limitations, the use of ERP in further studies is expected to increase in areas such as ASD and stress-related disorder, given that an association between ASD and ERP was revealed in this study.

## 5 Conclusion

These results indicate that ASD subjects have abnormalities in P300 compared with HCs. Moreover, the reduction in P300 could be a candidate neurophysiological marker for ASD.

## Author contributions

J-SC was responsible for the study concept and design. CH, MP, J-YL, HYJ, and SMP contributed to analysis of data and CH and MP wrote the manuscript. All authors contributed to and have approved the final manuscript.

**Conceptualization:** Jung-Seok Choi.

**Data curation:** Jun-Young Lee, Hee Yeon Jung.

**Formal analysis:** Minkyung Park, Su Mi Park.

**Funding acquisition:** Jung-Seok Choi.

**Investigation:** Jun-Young Lee, Hee Yeon Jung.

**Methodology:** Minkyung Park, Su Mi Park.

**Supervision:** Jun-Young Lee, Hee Yeon Jung, Jung-Seok Choi.

**Writing – original draft:** Changwoo Han, Minkyung Park.

**Writing – review & editing:** Jung-Seok Choi.
